# Visual Cycle Modulation as an Approach toward Preservation of Retinal Integrity

**DOI:** 10.1371/journal.pone.0124940

**Published:** 2015-05-13

**Authors:** Claes Bavik, Susan Hayes Henry, Yan Zhang, Kyoko Mitts, Tim McGinn, Ewa Budzynski, Andriy Pashko, Kuo Lee Lieu, Sheng Zhong, Bruce Blumberg, Vladimir Kuksa, Mark Orme, Ian Scott, Ahmad Fawzi, Ryo Kubota

**Affiliations:** 1 Acucela, Inc., 1301 2nd Avenue, Suite 1900, Seattle, Washington 98101, United States of America; 2 University of California, Irvine, School of Biological Sciences, 4351 Natural Sciences II, Irvine, California 92697, United States of America; University Zürich, SWITZERLAND

## Abstract

Increased exposure to blue or visible light, fluctuations in oxygen tension, and the excessive accumulation of toxic retinoid byproducts places a tremendous amount of stress on the retina. Reduction of visual chromophore biosynthesis may be an effective method to reduce the impact of these stressors and preserve retinal integrity. A class of non-retinoid, small molecule compounds that target key proteins of the visual cycle have been developed. The first candidate in this class of compounds, referred to as visual cycle modulators, is emixustat hydrochloride (emixustat). Here, we describe the effects of emixustat, an inhibitor of the visual cycle isomerase (RPE65), on visual cycle function and preservation of retinal integrity in animal models. Emixustat potently inhibited isomerase activity *in vitro* (IC_50_ = 4.4 nM) and was found to reduce the production of visual chromophore (11-*cis* retinal) in wild-type mice following a single oral dose (ED_50_ = 0.18 mg/kg). Measure of drug effect on the retina by electroretinography revealed a dose-dependent slowing of rod photoreceptor recovery (ED_50_ = 0.21 mg/kg) that was consistent with the pattern of visual chromophore reduction. In albino mice, emixustat was shown to be effective in preventing photoreceptor cell death caused by intense light exposure. Pre-treatment with a single dose of emixustat (0.3 mg/kg) provided a ~50% protective effect against light-induced photoreceptor cell loss, while higher doses (1–3 mg/kg) were nearly 100% effective. In Abca4-/- mice, an animal model of excessive lipofuscin and retinoid toxin (A2E) accumulation, chronic (3 month) emixustat treatment markedly reduced lipofuscin autofluorescence and reduced A2E levels by ~60% (ED_50_ = 0.47 mg/kg). Finally, in the retinopathy of prematurity rodent model, treatment with emixustat during the period of ischemia and reperfusion injury produced a ~30% reduction in retinal neovascularization (ED_50_ = 0.46mg/kg). These data demonstrate the ability of emixustat to modulate visual cycle activity and reduce pathology associated with various biochemical and environmental stressors in animal models. Other attributes of emixustat, such as oral bioavailability and target specificity make it an attractive candidate for clinical development in the treatment of retinal disease.

## Introduction

**Fig 1:**
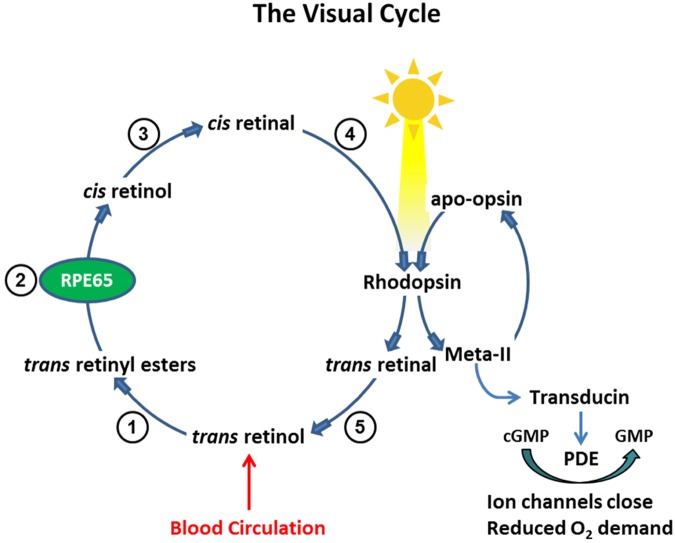
Processing of Vitamin A in The Visual Cycle. Enzymatic processing within the visual cycle begins with delivery of vitamin A (all-*trans*-retinol) from the blood circulation. Upon entry into the RPE, all-*trans*-retinol is converted to a retinyl ester through the activity of lecithin retinol acyl transferase (1). The resulting all-*trans*-retinyl ester pool represents a storage form of vitamin A upon which RPE65 acts to generate 11-*cis*-retinol (2); 11-*cis*-retinol is then oxidized by an 11-*cis*-specific retinol dehydrogenase to form the visual chromophore, 11-*cis*-retinal (3). The visual chromophore is delivered to rod and cone outer segments (4) where it combines with opsins to form visual pigments (e.g., rhodopsin). Light activation of rhodopsin initiates visual transduction processes and liberates all-*trans*-retinal as a photoproduct. Reduction of all-*trans*-retinal, via all-*trans*-retinal dehydrogenase, produces all-*trans*-retinol (5), which is transferred back to the RPE for recycling. The continued activity of RPE65 in the light state ensures sustained levels of rhodopsin, closure of ion channels through transducin activation, and reduced oxygen demand.

The conversion of dietary vitamin A (all-*trans*-retinol) into the light-sensitive visual chromophore (11-*cis*-retinal), and its regeneration following photobleaching, is accomplished through a series of enzymatic reactions known as the *visual cycle*. Processes of the visual cycle occur within the retinal pigment epithelium (RPE) and in outer segments of rod and cone photoreceptors (reviewed in [1]). Mobilization of stored vitamin A (all-*trans*-retinyl esters) is a key step of the visual cycle and is catalyzed by the RPE-specific protein, RPE65. This enzyme performs a unique hydrolysis/isomerization reaction to produce the visual chromophore precursor, 11-*cis*-retinol, from retinyl ester stores. Oxidation of 11-*cis*-retinol yields 11-*cis*-retinal, which is transferred from apical processes of the RPE to outer segments of rod and cone photoreceptors where it combines with the appropriate opsins to generate light-sensitive rod and cone visual pigments (Fig 1).

Photobleaching of the visual pigments liberates opsin apo-proteins and all-*trans*-retinal. Under normal physiological conditions, all-*trans*-retinal is reduced to all-*trans*-retinol and returned to the RPE for recycling. However, under conditions which allow all-*trans*-retinal to transiently accumulate in photoreceptor outer segments (e.g., during intense light exposure or due to a genetic defect), the retina becomes extremely susceptible to oxidative damage. All-*trans*-retinal is a toxic aldehyde and a potent photosensitizer [2,3]. It can induce lipid peroxidation through the generation of reactive oxygen species (ROS), such as singlet oxygen [4], and cause damage to outer segment proteins [5,6]. Because of its toxic properties, all-*trans*-retinal is believed to be a key mediator of retinal light damage [7]. Studies demonstrating that depletion of 11-*cis*-retinal by pharmacologic or genetic means provides protection against light-mediated retinal damage are consistent with this hypothesis [8,9,10].

Because all-*trans*-retinal is released in a phosphatidylethanolamine- (PE) rich environment, it is prone to a condensation reaction with the primary amine of PE to form a retinal-PE adduct [11]. A similar reaction is thought to occur with free 11-*cis*-retinal [12]. Retinal-PE adducts may undergo secondary condensation with additional retinal molecules leading to formation of bis-retinoid-PE complexes which can be processed to form stable amphiphilic compounds, such as *N*-retinylidene-*N*-retinylethanolamine (A2E) [13,14]. A2E is a well-characterized bisretinoid that has been shown to have several deleterious effects on RPE cells, including generation of ROS [15,16], impairment of lysosomal function [17,18], induction of pro-apoptotic proteins[19], complement activation [20], and upregulation of vascular endothelial growth factor (VEGF) [21]. In animal models of excessive A2E accumulation, compounds that either inhibit visual cycle activity or reduce vitamin A delivery to the RPE are effective to halt the accumulation of A2E and attenuate retinal pathology [22,23,24,25].

While prolonged or intense acute light exposure poses a potential risk for photo-oxidative retinal damage, dark adaptation engenders a significant cellular stress associated with high oxygen consumption [26,27]. During dark adaptation, photoreceptors consume large amounts of oxygen to fuel an ion pumping activity that is necessary to maintain electrochemical gradients across the outer segment membrane. In bright light, the channels through which the ions flow are closed, pumping activity ceases and retinal oxygen consumption decreases by 40%–60%, relative to the dark adapted state [28]. The high oxygen consumption of photoreceptors creates an extremely low oxygen tension in the outer retina generating a “sink” for any available oxygen, which can only come from the inner retina. In the context of an emerging, or compromised, inner retina vasculature, deprivation of retinal oxygen can create a hypoxic environment prompting the aberrant growth of new blood vessels to meet oxygen demands [29,30]. It has been theorized that prevention of complete dark adaptation, through activation of rod phototransduction during the dark cycle, may be effective to prevent hypoxia and preserve vasculature of the inner retina [31,32,33].

We have hypothesized that reduction of visual chromophore biosynthesis in the visual cycle could be an effective method to prevent retinal pathology caused by prominent cellular stressors such as light, retinoid byproducts, and oxygen. To evaluate this hypothesis, we have examined the effects of a specific inhibitor of RPE65 in diverse animal models which manifest retinal pathology caused by these stressors.

## Material and Methods

### Emixustat Hydrochloride

Emixustat hydrochloride (emixustat, chemical name: (*R*)-(+)-3-amino-1-(3-(cyclohexylmethoxy)phenyl)propan-1-ol hydrochloride) is a non-retinoid, small molecule with a molecular weight of 299.84 gram/mole (C_16_H_25_NO_2_•HCl). Chemical synthesis of emixustat is described in S1 Text.

### Mice

Mice (BALB/c and 129/Sv / C57BL/6 mixed background) were purchased from Charles River Laboratories International, Inc. (Wilmington, DE). Abca4 knockout mice were produced by inGenious Targeting Laboratory, Inc (Stony Brook, NY) as described below; these mice were transferred to Charles River where they were bred and maintained. All mice were kept in normal 12 hour light-dark cycle lighting, unless otherwise noted. Mice at the Charles Rivers facility were housed under ~485 lux light (~45 foot-candle) and were acclimated in our facility to cyclic light of ~300 lux for ≥3 days before starting experiments. Mice used in light damage studies were acclimated under ~300 lux light for ≥20 days. Manipulations during the dark periods and all analyses involving retinoids were carried out under dim red light. All procedures were approved by the Acucela Institutional Animal Care and Use Committee and were in compliance with the ARVO Statement for the Use of Animals in Ophthalmic and Vision Research. Acucela has been accredited by the Association for Assessment and Accreditation of Laboratory Animal Care (AAALAC) for compliance to policies that promote the humane treatment of animals.

### Expression of Visual Cycle Proteins for *In Vitro* Isomerase Assays

cDNAs for human *RPE65* (Origene Technologies Inc., Rockville, MD) and lecithin:retinol acyltransferase (*LRAT*; American Type Culture Collection, Manassas, VA) were cloned into the pVITRO4-neo-mcs vector (Origene Technologies Inc., Rockville, MD), transfected into HEK293H cells, and the stable cell line was further transformed with another expression vector carrying only *RPE65* (Origene), to enhance RPE65 expression. Expression was verified by immunoblotting with a mouse anti-RPE65 antibody (Abcam, Cambridge, MA) and by LRAT activity assay. Human cellular retinaldehyde-binding protein (CRALBP) cDNA was generated by RT-PCR from total RNA from an ARPE19 cell line (ATCC, Manassus, VA) and expressed in E. coli (pTrcHis2-TOPO TA vector; Invitrogen Corp., Carlsbad, CA). Recombinant CRALBP-His protein was purified using a Ni-Sepharose FPLC system (GE Healthcare Life Sciences, Uppsala, Sweden); purity and quantity were assessed by SDS-PAGE and BCA assay, respectively (Thermo Fisher Scientific Inc., Rockford, IL).

### *In vitro* Isomerase Assays

Assays were performed as described [23], using a homogenate of the HEK293H cell line expressing human RPE65 and LRAT, with 20 μM all-*trans*-retinol (Toronto Research Chemicals; Toronto, Canada) as substrate. Recombinant human CRALBP (80 μg/mL) was added to facilitate formation of 11-*cis*-retinol [23,34]. Reaction mixtures contained 10 mM Bis-Tris propane, pH 7.5, 0.5% BSA, and 1 mM NaPPi. Emixustat (in ethanol, 0.5% v/v) was added to the assay at final concentrations of 10^-5^ to 10^-10^ M; control samples contained the ethanol vehicle. After 1 hour at 37°C, reactions were terminated with methanol, extracted with heptane, dried with a centrifugal evaporator, and resuspended in 150 μL of heptane. Retinoids were analyzed by normal phase HPLC (flow rate = 1.5 mL/min; mobile phase: 0.16% isopropanol, 7.84% ethyl acetate, 6% dioxane, and 86% hexane; Agilent 1100 system (Agilent Technologies, Santa Clara, CA), using an Ultrasphere Silica column (5 μm, 4.6 mm x 250 mm). Identification of retinoid peaks was confirmed by on-line spectral analysis (210–450 nm).

### *In vitro* binding to retinoic acid nuclear receptors

The activity of emixustat (10^-5^ M—10^-11^ M) was evaluated in human retinoic acid receptors (RAR-α, -β, and -γ) and retinoic X receptor-alpha assays performed in triplicate as described^27^ using 9-*cis*-retinoic acid, and the synthetic retinoids TTNBP (Biomol Research Labs, Inc., Farmingdale, NY) and AGN203 (kind gift from R. Chandraratna, Allergan Inc., Irvine CA) as positive controls (10^-7^ M—10^-11^ M).

### Visual Chromophore Regeneration

Regeneration of 11-*cis*-retinal after photobleaching was assessed as described [22]. Dark-adapted (≥12 hours) BALB/c mice (8 weeks, n = 4/group) were orally dosed with emixustat (0.01–3.0 mg/kg) or vehicle (water). At 4 hours post-dose, animals were photobleached (5000 lux white light, 10 min) without anesthesia or mydriatics, returned to the dark for 2 hours, and then euthanized under red light. Enucleated eyes were homogenized in 10 mM Bis-Tris propane, pH 7.3 and 30 mM hydroxylamine, methanol and heptane were added, samples were vortexed and centrifuged (10 min, 13,200 rpm, 4°C). Retinoids in the supernatant were analyzed by normal phase HPLC (flow rate = 1.5 mL/min; mobile phase: 0.15% isopropanol, 14.85% ethyl acetate, and 85% hexane; Agilent 1100 system), with a silica column (Agilent Technologies, 4.6 mm x 250 mm, 5 μm). Identification of retinoid peaks was confirmed by on-line spectral analysis (210–450 nm). Retinal oxime levels from emixustat- and vehicle-treated mice were compared to determine the percent chromophore regeneration.

### Electroretinography

Recovery of rod photorecptor activity after photobleaching was assessed by electroretinography (ERG) as described [22,35], using the e^2^ Espion ERG system (Diagnosys, Lowell, MA). Dark-adapted (≥12 hours) BALB/c mice (8 weeks, n = 4/group) were orally dosed with emixustat (0.1–3 mg/kg) or vehicle (water). Mice remained in the dark and ERG measurements were performed 4 hours after treatment. Ten minutes before ERG measurements were made, mice were anesthetized with Ketamine:Xylazine (100:10 mg/kg) in PBS (intraperitoneal, i.p., injection; 4 μL/g). Left eyes were dilated with 1 drop of 0.5% tropicamide. All measurements were taken in a dark room under dim red light. Pre-bleach recordings in response to 0.01 cd*s/m^2^ (n = 5 measurements) were assessed after a 5 minute recovery from red light exposure. Mice were photobleached (400 cd/m^2^, 30 sec) and scotopic ERG b-wave responses (amplitudes in μV) to 0.01 cd*s/m^2^ stimuli were recorded every 2 minutes for 50 minutes. Analysis of rod a-wave recovery was performed in a similar manner using a lower emixustat dose range (up to 0.8 mg/kg) due to the smaller amplitude of the a-wave, relative to the b-wave.

### Light damage

The extent of light damage to the retina after exposure to intense white light was estimated from histological measurement of outer nuclear layer (ONL) thickness as described [36]. After dark adaptation overnight, BALB/c mice (4 mice/group) were treated with a single oral dose of emixustat (0.3–3 mg/kg) or vehicle (water). All groups, except the vehicle-treated dark control group, were treated with 0.5% tropicamide eye drops and subjected to light treatment (8,000 lux of white light for 1 hour) without anesthesia 4 hours post-dose. Mice not exposed to light served as negative (dark) controls. Mice were returned to recover overnight in the dark and ONL thickness was measured after two weeks in normal cyclic light.

Mice were euthanized with CO_2_, eyes were enucleated and marked to preserve orientation. The eyes were placed in Davidson’s fixative for overnight fixation followed by an overnight wash in 70% ethanol and were then embedded in paraffin. Tissue sections (5 μm) were cut along the vertical meridian of the eye to produce sections of central retina that extended from the superior to the inferior edge. The sections were deparaffinized in xylene and hydrated in an alcohol gradient. The sections were stained with hematoxylin/eosin and mounted. Sections were examined by light microscopy and photography (40 x 10 mag.). ONL measurements for all sections were obtained from comparable regions of the central retina. The number of ONL nuclei intersected by five lines perpendicular to the retina and evenly oriented was counted and the average cell number represented the ONL thickness for that eye.

### Accumulation of A2E and Lipofuscin Autofluorescence

ATP-binding cassette, sub-family A, member 4 (*Abca4*) knockout mice were generated as described;^30^ exon 1 of the *Abca4* gene was replaced with a Neo cassette (inGenious Targeting Laboratories Inc, Stony Brook, NY). Mice (mixed 129/Sv and C57BL/6 background) were then bred to homozygosity for the Leu450 variant of the *Rpe65* gene, to maximize accumulation of A2E.^31^ *Abca4* ^*-/-*^ mice 2 months of age were orally dosed for 3 months with 0.03–3 mg/kg/day emixustat (n = 6–8/group) or vehicle (water; n = 4/group). Untreated *Abca4* ^*-/-*^ mice (n = 6–8/group) were evaluated at study onset to provide baseline (Day 0) A2E levels. Mice in the emixustat treatment groups remained healthy and active throughout the treatment period. At the end of the dosing period, eyes were enucleated, homogenized in chloroform/methanol and A2E levels were analyzed by HPLC [37]. Histological analyses included four groups of mice: age- and strain-matched wild type mice orally dosed for 3 months with vehicle; *Abca4* ^*-/-*^ mice orally dosed for 3 months with vehicle; *Abca4* ^*-/-*^ mice orally dosed for 3 months with 0.3 mg/kg emixustat; and *Abca4* ^*-/-*^ mice orally dosed for 3 months with 3.0 mg/kg emixustat. At the end of the dosing period, eyes from these four groups of mice were enucleated, marked to preserve orientation, fixed (4% PFA) and frozen embedded in OCT media. Tissue sections were prepared from comparable regions near the central retina and autofluorescence was imaged using a FITC excitation filter.

### Oxygen-induced Retinopathy

The mouse model of oxygen-induced retinopathy (OIR) was used to evaluate effects of emixustat treatment on retinal neovascularization [38]. Seven day-old mouse pups (129/Sv; 10 litters of 5–7 pups per treatment condition) with nursing mothers were subjected to hyperoxia (75% oxygen) for 5 days. On P12, the mice were returned to room air and daily i.p. injections of ruboxistaurin (10 mg/kg, Axon Drugs Pvt. Ltd, India), ruboxistaurin vehicle (5% DMSO), emixustat (0.03–3.0 mg/kg), or emixustat vehicle (water) were administered over the ensuing 5 days. On day 17, the mice were sacrificed, eyes were enucleated and fixed in 4% PFA (2 hours at 4°C) and then transferred to cold PBS. Retinas were dissected from the eyeglobes, dehydrated in cold methanol (15 min) and washed in cold ICC buffer (0.5% BSA, 0.2% Tween20, 0.1% TritonX-100 in PBS). Retinas were stained with isolectin B4 in ICC buffer (1 μg/μL, overnight at 4°C), followed by a wash in cold ICC buffer. Four radial cuts were made along the peripheral retina to allow flat mounting. Retinas were prepared in mounting media for microscopic analysis of retinal neovascularization (NV) (Adobe Photoshop).

### Analyses

GraphPad Prism software (La Jolla, CA) was used to calculate IC_50_, EC_50_, and ED_50_ values; data were fit with sigmoidal-dose response curves (var. slope; 4 param. logistic equations). One-way ANOVA with Tukey multiple comparison tests was used for all statistical analyses.

## Results

### Inhibition of Isomerase Activity and Visual Chromophore Regeneration

**Fig 2:**
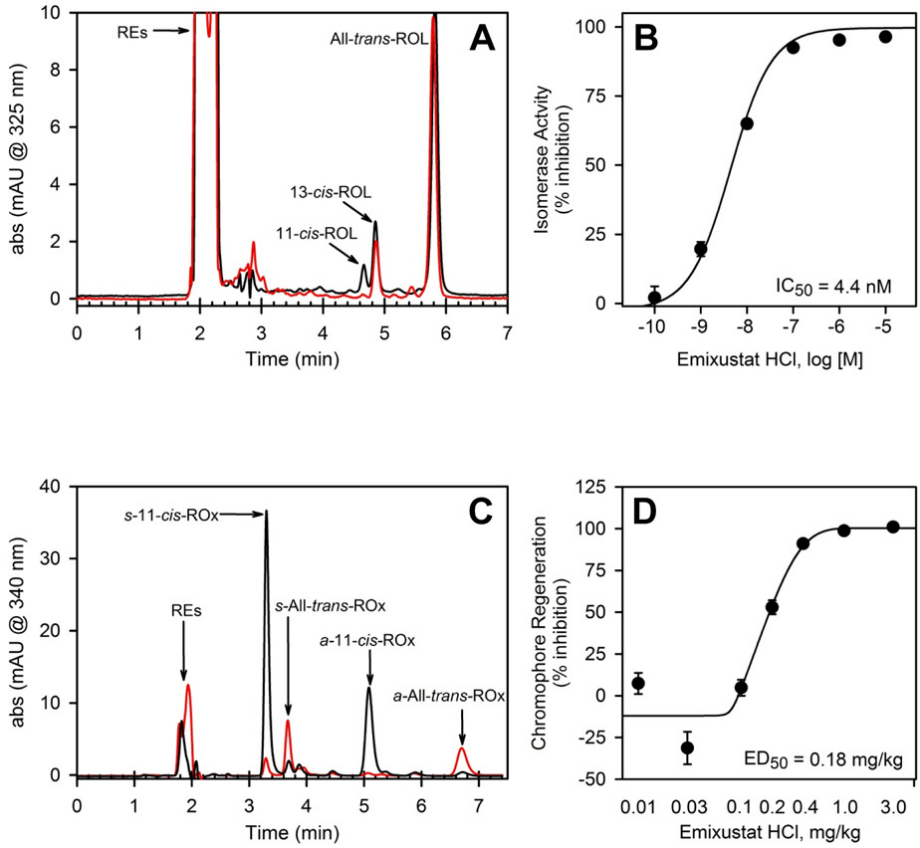
Inhibition of Isomerase Activity and Visual Chromophore Regeneration. The effect of emixustat on isomerase activity and visual chromophore regeneration was evaluated in *in vitro* and *in vivo* assays. Reaction conditions for *in vitro* assays are described in *Methods*. Retinoids in the sample extracts were measured by HPLC (RE, retinyl esters; ROL, retinols). Panel A shows representative HPLC chromatographic tracings from a control sample containing no emixustat (black trace) and a sample containing 1 μM emixustat (red trace). Note absence of the 11-*cis*-ROL peak in the sample containing emixustat. The dose-response curve for inhibition of 11-*cis*-ROL production by emixustat is provided in panel B (each data point represents mean ± SEM, n = 8). The mean 11-*cis*-ROL level in control samples (no emixustat) was taken as 0% inhibition; half maximal inhibitory concentration (IC_50_) for 11-*cis*-ROL production in the presence of emixustat is provided in the panel B inset. The effect of emixustat on visual chromophore regeneration *in vivo* was evaluated in wild-type mice as described in *Methods*. In these analyses, the amount of 11-*cis*-retinal production, at 2 hours in darkness following a photobleach (measured by HPLC as *syn-* and *anti*-11-*cis*-retinal oximes, *s*-11-*cis*-ROx and *a*-11-*cis*-ROx, respectively), in mice treated with varied doses of emixustat was examined. Sample chromatographic tracings of the retinoid profile from a vehicle-treated mouse (black trace) and a mouse treated with 1 mg/kg emixustat (red trace) are shown in panel C (*syn-* and *anti*-All-*trans*-retinal oximes, *s*-All-*trans*-ROx and *a*-All-*trans*-ROx, and all-*trans*-retinyl esters, REs, are also shown). Note reduced level of *syn-* and *anti*-11-*cis*-retinal oxime peaks and increased REs in the trace from the emixustat-treated mouse. The dose-response curve for inhibition of visual chromophore production by emixustat is provided in panel D (each data point represents mean ± SEM, n = 8). The mean 11-*cis*-retinal oxime level in control samples (no emixustat) was taken as 0% inhibition. The calculated half maximal effective dose (ED_50_) for inhibition of visual chromophore production by emixustat is provided in the panel D inset.

The potency of emixustat to inhibit isomerase activity was evaluated in an *in vitro* assay system. Homogenates from cells overexpressing RPE65 and LRAT were incubated with all-*trans*-retinol (all-*trans*-ROL), CRALBP, and increasing concentrations of emixustat. The production 11-*cis*-retinol (11-*cis*-ROL) was measured by HPLC (Fig 2A). The data showed a concentration-dependent reduction of 11-*cis*-ROL production (i.e., inhibition of RPE65 isomerase activity) by emixustat. Analysis of the 11-*cis*-ROL data gave an IC_50_ value of 4.4 ± 0.59 nM for inhibition of RPE65 isomerase activity (Fig 2B).

Inhibition of RPE65 isomerase activity *in vivo* would be expected to slow regeneration of visual chromophore (11-*cis-*retinal); this was assessed by measuring the extent of 11-*cis-*retinal regeneration (measured as *syn-* and *anti*-11-*cis*-retinal oximes) during recovery in darkness following a photobleach. Dark-adapted mice were treated with emixustat (0.01–3 mg/kg), photobleached 4 hours after dosing, and returned to darkness for 2 hours to allow regeneration of 11-*cis*-retinal. The HPLC data showed a dose-dependent reduction of 11-*cis*- and all-*trans*-retinal oximes in mice treated with emixustat. The data also showed an accumulation of retinyl esters in emixustat-treated mice, consistent with reduced mobilization of this storage pool due to RPE65 inhibition (Fig 2C). The ED_50_ for the effect of emixustat on inhibition of 11-*cis*-retinal oxime production was determined to be 0.18 mg/kg (Fig 2D). Importantly, the effect of emixustat was reversible; 11-*cis*-retinal oxime levels returned to near control levels by 24 hours after dosing.

### Activation of Retinoic Acid Receptors

Although emixustat is not a retinoid and, therefore, should neither bind nor activate retinoic acid receptors, it can affect retinoid levels in the visual cycle. Therefore, we examined the effect of emixustat on RAR and RXR binding/activation *in vitro*. The positive controls (TTNBP, AGN203) activated RAR and RXR as expected. However, no activation was observed with emixustat at concentrations from 10^-5^ to 10^-11^ M (data not shown).

### Pharmacodynamic Effect

**Fig 3:**
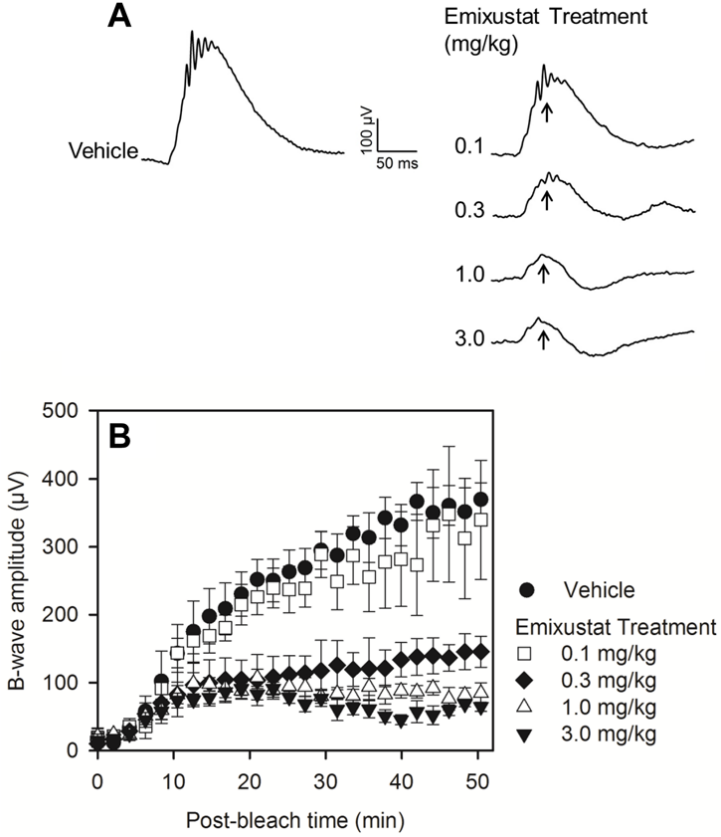
Pharmacodynamic Effect of Emixustat on Rod Function. The recovery of rod photoreceptor function following a photobleach was measured by ERG. Mice were treated with varied doses of emixustat (n = 4/dose group), or vehicle (n = 4/group), and ERG responses were recorded every 2 minutes for 50 minutes using a light stimulus 0.01 cd*s/m^2^. Panel A shows representative ERG waveforms in vehicle- and emixustat-treated mice. Arrows in panel A indicate the b-wave amplitude peaks which are plotted as a function of recovery time after photobleach in panel B. B-wave response amplitudes (μV), at each corresponding time point of recovery, are shown as mean values ± SEMfor each of the treatment groups (panel B). Emixustat treatment caused a dose-dependent suppression of b-wave response amplitudes. The dose required for half-maximal suppression of ERG b-wave recovery was determined to be 0.21 mg/kg.

The effect of emixustat on the physiological response of the retina to light was assessed by evaluating recovery of rod photoreceptor function by ERG after photobleaching (Fig 3). Dark adapted mice were treated with increasing doses of emixustat (0.1–3 mg/kg) or vehicle; 4 hours after dosing, animals were photobleached (400 cd/m^2^, 30 sec) and recovery of rod function was recorded using scotopic stimuli (0.01 cd*s/m^2^). Representative ERG traces obtained from mice in each treatment condition are shown in Fig 3A. In vehicle-treated mice, b-wave amplitudes were robust and recovered to pre-bleach values within 40–50 minutes. Meanwhile, in mice treated with emixustat, b-wave response amplitudes were diminished and recovery was slowed in a dose-dependent manner (Fig 3B). The dose associated with half-maximal suppression of rod b-wave recovery (ED_50_) was determined to be 0.21 mg/kg. Calculation of the ED_50_ for a-wave recovery gave a value of 0.18 mg/kg, which was comparable to the b-wave ED_50_ (not shown). Suppression of rod photoreceptor amplitude recovery induced by emixustat was reversible; between 16 to 24 hours after dosing, the rate of amplitude recovery equaled or exceeded the rate in vehicle-treated mice (data not shown).

### Light Damage

**Fig 4:**
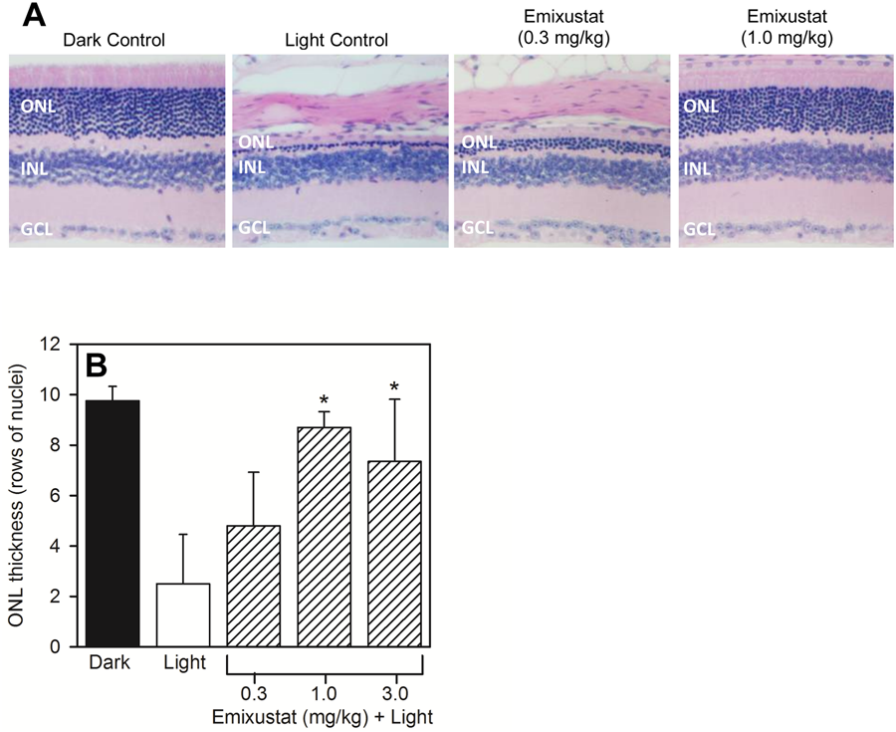
Protection from Light Damage. The ability of emixustat to provide protection from light damage was assessed as described in *Methods*. Mice received a single dose of emixustat, or vehicle, prior to light exposure (8,000 lux white light, 1 hour). Histological analyses and determination of ONL thickness was performed following a 2-week recovery period. Panel A shows tissue sections prepared from untreated, dark-adapted mice, untreated light-exposed mice, and mice pre-treated with either 0.3 or 1.0 mg/kg emixustat (ONL, outer nuclear layer; INL, inner nuclear layer; GCL, ganglion cell layer). Quantitative analysis of ONL layer thickness (panel B) was used to calculate the ED_50_ for preservation of the ONL in emixustat-treated mice (ED_50_ = 0.20 mg/kg). There was a statistically significant preservation of ONL thickness in mice treated with 1.0 or 3.0 mg/kg emixustat compared to the vehicle-treated, light control group (*, p<0.01).

Albino (BALB/c) mice were used to assess the effects of emixustat on protection from light damage. Dark adapted, untreated albino mice showed a healthy retinal morphology with ~10 rows of nuclei in the ONL (Fig 4A, Dark Control). Exposure to 8,000 lux white light for 1 hour caused near complete destruction of the ONL in untreated mice (Fig 4A, Light Control). Pre-treatment with a single dose of emixustat provided dose-dependent protection of the ONL (Fig 4A, Emixustat 0.3 mg/kg and 1.0 mg/kg). Measurement of the ONL thickness (number of nuclei) showed a reduction from 10 nuclei in the Dark Control to 2.5 nuclei in the Light Control (Fig 4B). In contrast, ONL thickness in mice pre-treated with emixustat was well preserved. Mice treated with either 1.0 or 3.0 mg/kg emixustat showed a statistically significant thicker ONL compared to the vehicle treated group (t-test, p<0.01). The calculated ED_50_ for preservation of the ONL in emixustat-treated mice was 0.20 mg/kg.

### Accumulation of A2E and Lipofuscin Autofluorescence

**Fig 5:**
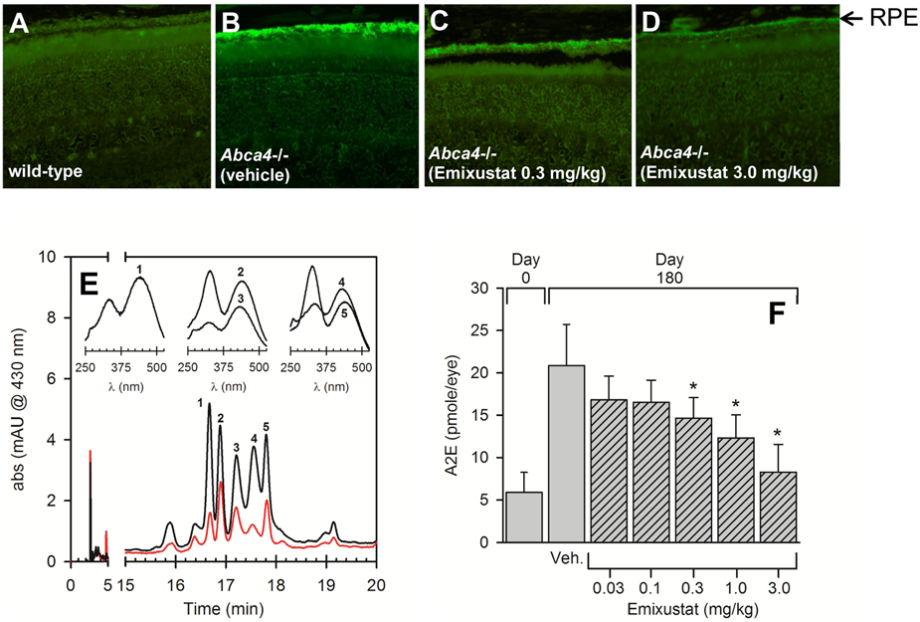
Reduced Lipofuscin Autofluorescence and A2E Accumulation. The effect of emixustat on lipofuscin autofluorescence and A2E levels was examined in an animal model of autosomal recessive Stargardt disease (*Abca4* ^-/-^ mice). Histological analysis of lipofuscin autofluorescence in untreated, strain- and age-matched wild-type mice, and *Abca4* ^-/-^ mice treated with either vehicle or emixustat (0.3 or 3.0 mg/kg) is shown in panels A—D, respectively. All mice were 5 months of age. Lipofuscin fluorophores were extracted from RPE eyecups and analyzed by HPLC as described in *Methods*. Representative chromatograms from eyecup extracts of mice treated with emixustat (3 mg/kg for 3 months) and vehicle (red and black tracings, respectively) are shown in panel E. UV-vis spectra associated with the indicated peaks (numbered 1–5) are shown in the panel inset. Peak 1 in the chromatogram was determined to be A2E based upon spectral identity and co-elution with an authentic A2E standard. Quantitative analysis of A2E levels (based on area units of peak 1) in *Abca4* ^-/-^ mice is shown in panel F (peaks 2–4 were not quantified). Numbers of mice analyzed for A2E quantitation are as follows: Day 0: n = 17; Vehicle: n = 6; 0.03 mg/kg: n = 6; 0.1 mg/kg: n = 7; 0.3 mg/kg, n = 8 1.0 mg/kg, n = 8; 3 mg/kg, n = 8. A2E levels increased from ~5 to ~20 pmoles/eye over a 3-month period in vehicle-treated *Abca4* ^-/-^ mice. *Abca4* ^-/-^ mice treated with emixustat showed a dose-dependent reduction of A2E which was statistically significant at doses ≥ 0.30 mg/kg/day, relative to vehicle treated *Abca4* ^-/-^ mice (*, p<0.05). The ED_50_ for the effect of emixustat on reducing accumulation of A2E was 0.47 mg/kg/day.

The effect of emixustat on the accumulation of A2E and lipofuscin autofluorescence was examined in *Abca4* ^-/-^ mice, an animal model of autosomal recessive Stargardt disease. The protein encoded by the *Abca4* gene facilitates transport of retinaldehyde-PE complexes across the rod outer segment disc membrane, which allows for efficient recycling of visual cycle retinoids and reduces the accumulation of retinal derivatives such as A2E [14,39]. Compared to age- and strain-matched wild-type mice, the RPE of *Abca4* ^-/-^ mice shows a pronounced autofluorescence due to the accumulation of A2E and related compounds (compare Fig 5A and 5B). Treatment of *Abca4* ^-/-^ mice with emixustat (3 months daily treatment, 0.3 or 3 mg/kg/day) markedly reduced RPE autofluorescence (Fig 5C and 5D, respectively). Analysis of A2E levels in all groups of *Abca4* ^-/-^ mice (2 month old controls, and 5 month old mice treated with either vehicle or emixustat) revealed a dose-dependent reduction of A2E (Fig 5E). Emixustat doses above 0.03 mg/kg/day produced a statistically significant reduction of A2E compared to vehicle-treated *Abca4* ^-/-^ mice (p<0.001). The ED_50_ for the effect of emixustat on reducing accumulation of A2E was 0.47 mg/kg/day.

### Oxygen-induced Retinopathy

**Fig 6:**
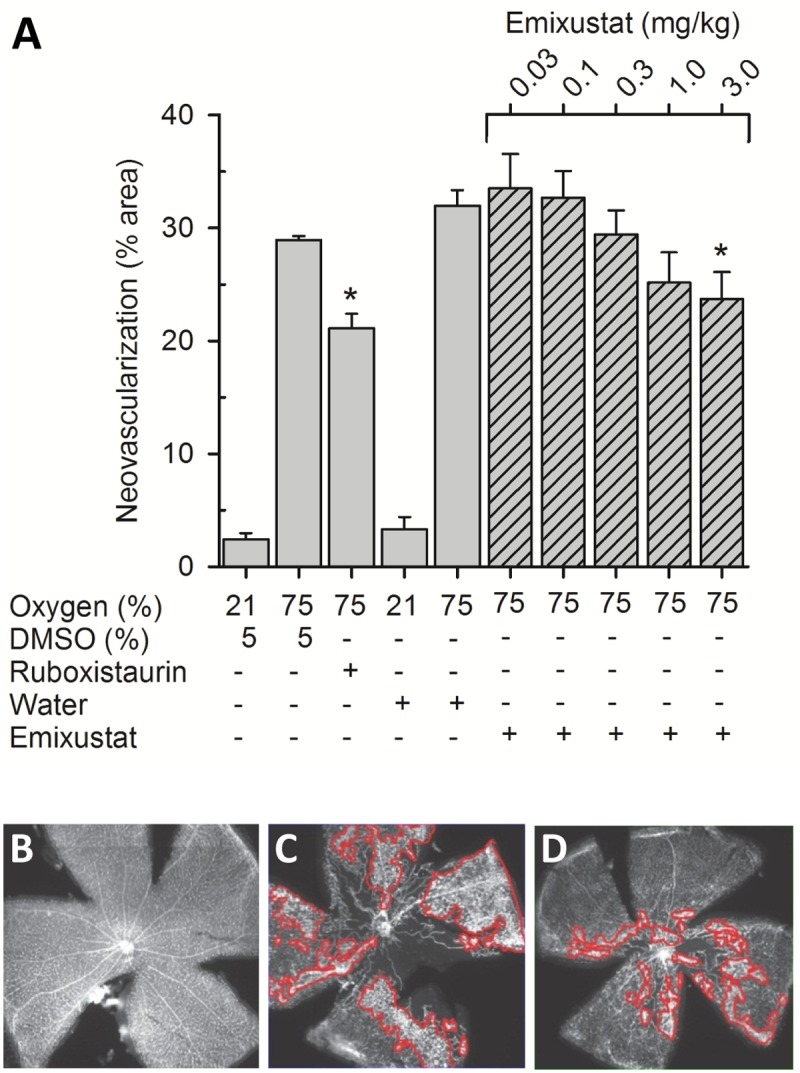
Reduced Retinal Neovascularization. The effect of emixustat on retinal neovascularization was studied in the mouse OIR model. Seven day-old mouse pups were subjected to hyperoxia (75% oxygen) for 5 days. On P12, the mice were returned to room air and daily treatments with ruboxistaurin (10 mg/kg), emixustat (0.03–3.0 mg/kg), or appropriate vehicles were administered as described in *Methods*. Retinal flat mounts were prepared and areas of NV were quantified; these data were compared to data from control mice that were maintained in a normoxic environment (21% oxygen). Mice that were moved from a hyperoxic to normoxic environment, without treatment, showed a significant extent of retinal NV (~30% of the retinal area). Treatment with the ruboxistaurin (positive control) reduced the area of NV to ~20% of the total retinal area. In mice treated with emixustat, a dose-dependent reduction in retinal NV was observed. The reduction in NV at the highest emixustat dose (3.0 mg/kg/day) approached a level that was comparable to that obtained with ruboxistaurin (Fig 6A; *, t-test, p<0.05). The ED_50_ for reduction of retinal NV in emixustat-treated mice was 0.46 mg/kg/day. Representative retinal flat mounts from an untreated, normoxic control, an untreated OIR control, and in a 3 mg/kg emixustat-treated mouse are shown in panels B, C, and D, respectively. Areas of NV, outlined in red tracings, were identified and quantified using Adobe Photoshop software.

We hypothesized that reduced chromophore biosynthesis might be effective to prevent complete dark adaptation and, thus, may potentially reduce oxygen consumption in the inner retina and preserve integrity of the retinal vasculature. To explore this possibility, we administered emixustat (0.03–3 mg/kg) to OIR mice during the period of reperfusion injury (P12—P17) and examined the extent of retinal NV in flat mounted retinas. Ruboxistaurin, a PKCß inhibitor that is known to inhibit VEGF-mediated retinal NV, was included a positive treatment control. Daily administration of emixustat during P12-P17 produced a dose-dependent reduction in retinal NV. The 3 mg/kg/day dose of emixustat reduced the extent of retinal NV by ~30%, which was comparable to reduction observed with a 10 mg/kg ruboxistaurin (Fig 6A). The ED_50_ for reduction of retinal NV in emixustat-treated mice was 0.46 mg/kg. Representative retinal flat mounts from an untreated, normoxic control, an untreated OIR control, and in a 3 mg/kg emixustat-treated mouse are shown in panels B, C, and D, respectively.

## Discussion

The objective of the present study was to evaluate reduction of visual chromophore biosynthesis as a means to preserve retinal integrity in animal models of human retinal pathology. We were particularly interested in photooxidative stress, accumulation of retinoid byproducts and retinal hypoxia as these are suspected to be prominent mediators of photoreceptor damage and degeneration in human retinal diseases (e.g., age-related macular degeneration (AMD), Stargardt disease, proliferative diabetic retinopathy, and diabetic macular edema). Our approach was to develop an oral non-retinoid, small molecule inhibitor of RPE65 (emixustat) and assess treatment effects in mouse models of light-mediated retinal damage, aberrant lipofuscin accumulation, and oxygen-induced retinopathy. Biochemical and electrophysiological analyses of visual chromophore biosynthesis demonstrated that emixustat potently inhibits RPE65 in a dose-dependent, reversible manner. In each model studied, emixustat treatment either improved the anatomical phenotype or completely preserved photoreceptor integrity. Notably, the dose required to produce 50% of the maximal treatment effect (ED_50_) in each of the models studied was comparable (0.20–0.47 mg/kg) and was similar to the ED_50_ values determined for reduction of visual chromophore biosynthesis (ED_50_ = 0.18 mg/kg) and the pharmacodynamic effect of emixustat (reduction in rod photoreceptor recovery following a photobleach, ED_50_ = 0.21 mg/kg). These relationships suggest that inhibition of RPE65 by emixustat, may be the primary mechanism of action underlying the observed treatment effects.

Inhibition of visual cycle function as a potential therapeutic approach for the treatment of retinal disease, or preservation of retinal function, has been an active area of study during the past several years. Previous investigations have focused primarily on reduction of lipofuscin fluorophores and/or protection from light damage as pre-clinical efficacy measurements using retinoid-based compounds [4,7,9,25,40]. Of particular note are the systematic studies of retinylamine (Ret-NH_2_) conducted by Palczewski and coworkers [4,9,40]. Originally developed as a transition state analog for the RPE65 isomerization reaction, Ret-NH_2_ was shown to be a potent inhibitor of RPE65 [22,23]. The therapeutic potential of Ret-NH_2_ was examined in a line of mice which rapidly accumulated all-*trans*-retinal condensation products due to a null mutation in the *Abca4* and retinol dehydrogenase 8 (*Rdh8*) genes. The aberrant phenotype of these mice, which included early accumulation of lipofuscin/A2E, drusen, basal laminar deposits, Bruch's membrane thickening, and choroidal neovascularization, was significantly reduced following treatment with Ret-NH_2_ [4,40]. These findings provided additional evidence for the link between accumulation of retinaldehyde toxins and RPE/photoreceptor degeneration and demonstrated that pharmacologic intervention in the visual cycle could be a tractable therapeutic approach.

Emixustat possesses important pharmacologic attributes which distinguish it from other small molecule RPE65 inhibitors. First, emixustat is a non-retinoid compound. As such, it cannot be metabolized to form a retinoid and is shown in the present study to have no activity against retinoic acid receptors. Secondly, emixustat demonstrates an extremely high and selective inhibitory potency against RPE65 isomerase activity (IC_50_ = 4.4 nM), providing the potential for less drug exposure in chronic dosing regimens. Finally, we have observed an anti-angiogenic effect of emixustat which has not been previously demonstrated with other RPE65 inhibitors, but is explainable based upon the mechanism of emixustat action.

High oxygen consumption of photoreceptors during dark adaptation is believed to produce ischemia within the inner retina promoting aberrant new vessel growth. In the light state, activation of phototransduction significantly reduces oxygen demand of photoreceptors. Meta-II-mediated activation of transducin is key in this process. Importantly, apo-opsin is also capable of activating transducin and can produce an adaptation state much like that produced by real light [41,42,43]. Treatment with emixustat specifically reduces visual chromophore levels without affecting levels of the opsin protein. Thus, it is conceivable that during emixustat treatment, apo-opsin could accumulate in amounts sufficient to stimulate phototransduction which would decrease oxygen demand during the dark state and alleviate inner retinal ischemia. This effect would explain reduced retinal NV in OIR mice treated with emixustat.

In summary, data from the present study provide strong evidence that reduction of visual chromophore biosynthesis is an effective method to alleviate photoreceptor cell stress and preserve retinal integrity. The ability of emixustat to specifically target RPE65 and attenuate retinal pathology elicited by three prominent and distinct cellular stressors provides an impetus to explore therapeutic approaches in the treatment of degenerative retinal diseases in which these stressors have been implicated.

## Supporting Information

S1 Text Synthesis of Emixustat Hydrochloride. (DOCX)
