# Supplementary material for: Visual Cycle Modulation as an Approach toward Preservation of Retinal Integrity
Source: PLoS One. 2015 May 13;10(5):e0124940. doi: 10.1371/journal.pone.0124940 (PMC4430241; doi:10.1371/journal.pone.0124940)
Supplement: S1 Text — (DOCX) [file pone.0124940.s001.docx]

**Supplementary Material**

*Synthesis of Emixustat Hydrochloride*

Reagents and solvents were used as received from commercial suppliers. Anhydrous solvents were used for synthetic transformations generally considered moisture sensitive. Flash column chromatography and thin layer chromatography (TLC) were performed on silica gel. Gradient flash column chromatography was performed on a Biotage instrument. Proton nuclear magnetic resonance spectra were obtained on a Varian 400 spectrometer at 400 MHz for proton. Chemical shifts are given in ppm and coupling constants J are reported in Hertz (Hz). Residual protonated solvent was used as the reference peak for proton spectra. Reverse phase HPLC was performed on an Agilent HP1100 system with DAD detection at 220 nm using water-acetonitrile gradients with 0.05% trifluoroacetic acid. Mass-spectra were recorded using electrospray ionization mode (ES+) on an Agilent MSD mass spectrometer detector. Chiral HPLC analysis was performed using Chiralpak IA columns (4.6 x 250 mm, 5µm) on an Agilent HP1100 system with DAD detection at 220 nm using heptane-EtOH with 0.1% ethanesulfonic acid as the solvent.

Two synthetic methods were used to prepare emixustat used in the present study. Each method yielded an identical structural form of emixustat with similar *in vitro* and *in vivo* activity against the intended target (RPE65).

*Method 1* -The Mitsunobu reaction [[1](#_ENREF_1)] between 3-hydroxybenzaldehyde and cyclohexylmethanol gave 3-(cyclo-hexylmethoxy)benzaldehyde after purification by flash chromatography. Addition of acetonitrile to the aldehyde in the presence of lithium diisopropylamide gave the hydroxynitrile which was subsequently reduced with lithium aluminum hydride to *rac*-3-amino-1-(3-(cyclohexyl-methoxy)phenyl)propan-1-ol. Fmoc [[2](#_ENREF_2)] protection of the above amine followed by MnO_2_-mediated oxidation of the secondary benzylic alcohol afforded (9*H*-fluoren-9-yl)methyl (3-(3-(cyclohexylmethoxy)phenyl)-3-oxopropyl)carbamate. Stereoselective reduction of the ketone with (-)-*B*-chlorodiisopinocampheylborane yielded (*R*)-(9*H*-fluoren-9-yl)methyl (3-(3-(cyclohexylmethoxy)phenyl)-3-hydroxypropyl)carbamate with 96.9% chiral purity (93.8% ee). DBU (1,8-Diazabicyclo[5.4.0]undec-7-ene) deprotection of the carbamate followed by flash chromatography purification afforded emixustat free base as a colorless oil. ^1^H NMR (400 MHz, CDCl_3_)  7.22 (t, *J*=8.0 Hz, 1H), 6.95 (t, *J*=1.6 Hz, 1H), 6.90 (d, *J*=7.6 Hz, 1H), 6.77 (ddd, *J*=8.0, 2.4, 0.8 Hz, 1H), 4.90 (dd, *J*=8.8, 3.2 Hz, 1H), 3.75 (d, *J*=6.4 Hz, 2H), 3.12 (br.s, 2H), 3.06 (ddd, *J*=12.4, 6.0, 4.0 Hz, 1H), 2.90-2.96 (m, 1H), 1.82-1.89 (m, 3H), 1.67-1.81 (m, 6H), 1.15-1.34 (m, 3H), 0.99-1.09 (m, 2H). LC-MS m/z 264.2 a.u.

*Method 2* - Alkylation of 3-hydroxybenzaldehyde with (bromomethyl)cyclohexane under basic conditions gave 3-(cyclohexylmethoxy)benzaldehyde. Acetonitrile addition in the presence of *t*-BuO^-^K^+^ gave the hydroxynitrile which was subsequently reduced with borane dimethylsulfide complex in THF to *rac*-3-amino-1-(3-(cyclohexylmethoxy)phenyl)propan-1-ol. Chiral resolution of the racemic amine with (*R*)-mandelic acid in *tert-*butyl methyl ether gave crude (*R*)-3-amino-1-(3-(cyclohexylmethoxy)­phenyl)­propan-1-ol (*R*)-mandelate monohydrate which was further purified by recrystallization from water:isopropanol (9:1). The (*R*)-mandelate salt was treated with aqueous NaOH in organic solvent and converted into hydrochloride salt by treatment with HCl/isopropanol solution in isopropyl acetate. (*R*)-(+)-3-Amino-1-(3-(cyclohexylmethoxy)­phenyl)propan-1-ol hydrochloride (emixustat hydrochloride) was obtained as a white solid with >98% ee. m.p. 171-172 °C. ^1^H NMR (400 MHz, DMSO-*d*_6_)  7.96 (br.s, 3H), 7.20 (t, *J*=7.9 Hz, 1H), 6.82-6.92 (m, 2H), 6.77 (ddd, *J*=7.9, 2.3, 0.9 Hz, 1H), 5.52 (br.s), 4.64 (dd, *J*=8.3, 4.4 Hz, 1H), 3.73 (d, *J*=6.2 Hz, 2H), 2.74-2.85 (m, 2H), 1.74-1.93 (m, 4H), 1.59-1.74 (m, 4H), 1.08-1.29 (m, 3H), 0.80-1.08 (m, 2H). α^25^_D_= + 31.0° (c 1.08, EtOH).

**References**

1. Mitsunobu O (1981) The Use of Diethyl Azodicarboxylate and Triphenylphosphine in Synthesis and Transformation of Natural Products. Synthesis 1: 1-28.

2. Carpino LAH, Grace Y. (1972) 9-Fluorenylmethoxycarbonyl amino-protecting group. . The Journal of Organic Chemistry 37: 3404.
